# Identical sequences found in distant genomes reveal frequent horizontal transfer across the bacterial domain

**DOI:** 10.7554/eLife.62719

**Published:** 2021-06-14

**Authors:** Michael Sheinman, Ksenia Arkhipova, Peter F Arndt, Bas E Dutilh, Rutger Hermsen, Florian Massip

**Affiliations:** 1 Theoretical Biology and Bioinformatics, Biology Department, Utrecht UniversityUtrechtNetherlands; 2 Division of Molecular Carcinogenesis, the Netherlands Cancer InstituteAmsterdamNetherlands; 3 Max Planck Institute for Molecular GeneticsBerlinGermany; 4 Berlin Institute for Medical Systems Biology, Max Delbrück CenterBerlinGermany; 5 Université de Lyon, Université Lyon 1, CNRS, Laboratoire de Biométrie et Biologie Evolutive UMR 5558VillleurbanneFrance; University of BaselSwitzerland; National Institute of Child Health and Human DevelopmentUnited States

**Keywords:** horizontal gene transfer, alignment-free method, antibiotic resistance, power law, genome evolution, Other

## Abstract

Horizontal gene transfer (HGT) is an essential force in microbial evolution. Despite detailed studies on a variety of systems, a global picture of HGT in the microbial world is still missing. Here, we exploit that HGT creates long identical DNA sequences in the genomes of distant species, which can be found efficiently using alignment-free methods. Our pairwise analysis of 93,481 bacterial genomes identified 138,273 HGT events. We developed a model to explain their statistical properties as well as estimate the transfer rate between pairs of taxa. This reveals that long-distance HGT is frequent: our results indicate that HGT between species from different phyla has occurred in at least 8% of the species. Finally, our results confirm that the function of sequences strongly impacts their transfer rate, which varies by more than three orders of magnitude between different functional categories. Overall, we provide a comprehensive view of HGT, illuminating a fundamental process driving bacterial evolution.

## Introduction

Microbial genomes are subject to loss and gain of genetic material from other microorganisms (Boto, 2010; Puigbò et al., 2014), via a variety of mechanisms: conjugation, transduction, and transformation, collectively known as horizontal gene transfer (HGT) (Soucy et al., 2015; García-Aljaro et al., 2017). The exchange of genetic material is a key driver of microbial evolution that allows rapid adaptation to local niches (Boucher et al., 2011). Gene acquisition via HGT can provide microbes with adaptive traits that confer a selective advantage in particular conditions (Koonin, 2016; Massey and Wilson, 2017) and eliminate deleterious mutations, resolving the paradox of Muller’s ratchet (Takeuchi et al., 2014). In addition, HGT could also facilitate DNA repair, the fixation of beneficial mutations and the elimination of costly mobile genetic elements such as phages or conjugative elements (see Ambur et al., 2016 and references therein).

Since the discovery of HGT more than 50 years ago (Freeman, 1951), many cases of HGT have been intensively studied. Several methods have been developed to infer HGT. Some methods rely on identifying shifts in (oligo-)nucleotide composition along genomes (Ravenhall et al., 2015). Clonal frame-based methods instead perform phylogenetic analysis on similar set of strains to identify recombination events (Croucher et al., 2015; Didelot and Falush, 2007). Other methods are based on discrepancies between gene and species distances, that is, surprising similarity between genomic regions belonging to distant organisms that cannot be satisfactorily explained by their conservation (Lawrence and Hartl, 1992; Nelson et al., 1999; Koonin et al., 2001; Novichkov et al., 2004; Dessimoz et al., 2008; Dixit et al., 2015; Caro-Quintero and Konstantinidis, 2015). For example, genomes from different genera are typically up to 60-70% identical, meaning that one in every three base pairs is expected to differ. The presence of regions that are significantly more similar than expected can be interpreted as evidence of recent HGT events. Using such methods, the transfer of drug and metal resistance genes (Huddleston, 2014), toxin-antitoxin systems (Van Melderen and Saavedra De Bast, 2009), and virulence factors Escobar-Páramo et al., 2004; Nogueira et al., 2009 have been observed numerous times. It is also known that some bacterial taxa, such as members of the family of Enterobacteriaceae (Doi et al., 2012), are frequently involved in HGT, whereas other groups, such as extracellular pathogens from the *Mycobacterium* genus (Eldholm and Balloux, 2016), rarely are. Notably, the methods used in the detection and analysis of instances of HGT are computationally complex and can be used to discover HGT events in at most hundreds of genomes simultaneously. Consequently, a general overview of the diversity and abundance of transferred functions, as well as the extent of involvement across all known bacterial taxa in HGT, is still lacking. In particular, exchanges of genetic material between distant species – because discovering such long-distance transfers requires the application of computationally costly methods to very large numbers of genomes – are rarely studied.

In this study we use a novel approach to address these questions and challenges. Our method is based on the analysis of long exact sequence matches found in the genomes of distant bacteria. Exact matches can be identified very efficiently using alignment-free algorithms (Delcher et al., 2002), which makes the method much faster than previous methods that rely on alignment tools. We identified all long exact matches shared between bacterial genomes from different genera (see Identification of exact matches in Materials and methods). Such long matches are unlikely to be vertically inherited, and we therefore assume that they result from HGT. This allowed us to study transfer events between 1,343,042 bacterial contigs, belonging to 93,481 genomes, encompassing a total of 0.4 Tbp.

In a quarter of all bacterial genomes, we detected HGT across family borders, and 8% participated in HGT across phyla. This shows that genetic material frequently crosses borders between distant taxonomic units. The length distribution of exact matches can be accounted for by a simple model that assumes that exact matches are continuously produced by transfer of genetic material and subsequently degraded by mutation. Fitting this model to empirical data, we estimate the effective rate at which HGT generates long sequence matches in distant organisms. Furthermore, the large number of transfer events identified allows us to conduct a functional analysis of horizontally transferred genes.

## Results

### HGT detection using exact sequence matches

We identified HGT events between distant bacterial taxa by detecting long exact sequence matches shared by pairs of genomes belonging to different genera. We exploit that pairs of genomes from different genera are phylogenetically distant, so that sequences shared by both genomes due to linear descent (orthologous sequences) have low sequence identity. Therefore, long sequence matches in such orthologs are exceedingly rare. Generally, even the most conserved sequences in bacterial genomes from different genera have a nucleotide sequence identity of at most 90-95% (Qin et al., 2014). In the absence of HGT, the probability of observing an exact match longer than 300 bp between such regions in a given pair of genomes is then extremely small ($≃0.9^{300}≃10^{-14}$). Thus, even if millions of genome pairs with such divergence are analysed, the probability to observe even one long exact match in orthologous sequences remains negligible: one does not expect to find a single hit of this size between any two bacterial genomes.

**Figure 1.**
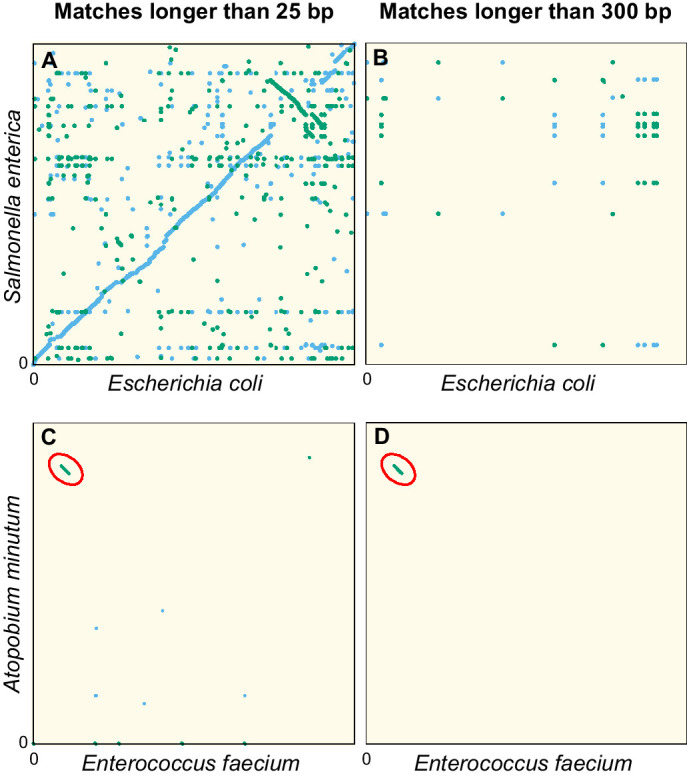
Dot plots of the exact sequence matches found in pairs of distant bacteria. On panels (**A** and **B**) resp. (**C and D**), each dot/line on the grid represents an exact match at locus x of the genome of *Escherichia coli* (resp. *Enterococcus faecium*) and locus y of the genome of *Salmonella enterica* (resp. *Atopobium minutum*). Blue dots/lines indicate matches between the forward strands of the two species, and green dots/lines those between the forward strand of *E. coli* (resp. *E. faecium*) and the reverse complement strand of *S. enterica* (resp. *A. minutum*). (**A–B**) Full genomes of *E. coli K-12 substr. MG1655* (U00096.3) and *S. enterica* (NC_003198.1), which both belong to the family of Enterobacteriaceae. Panel A shows all matches longer than 25 bp. The sequence similarity and synteny of both genomes, by descent, is evident from the diagonal blue line. Panel B only shows matches longer than 300 bp. (**C–D**) Same as panels (**A-B**), but for the first 1.4 Mbp of *E. faecium* (NZ_CP013009.1) and *A. minutum* (NZ_KB822533.1), which belong to different phyla, showing few matches longer than 25 bp (panel **C**). Yet, a single match of 19,117 bp is found, as indicated with red ellipses in panels (**C-D**). The most parsimonious explanation for this long match is an event of horizontal gene transfer.

Figure 1 illustrates this point. In the dot plot comparing the genome sequences of two Enterobacteriaceae, *Escherichia coli* and *Salmonella enterica* (Figure 1A), we observe numerous exact matches shorter than 300 bp along the diagonal, revealing a conservation of the genomic architecture at the family level. Filtering out matches shorter than 300 bp (Figure 1B) completely removes the diagonal line, confirming that exact matches in the orthologous sequences of these genomes are invariably short.

**Figure 1—figure supplement 1.**
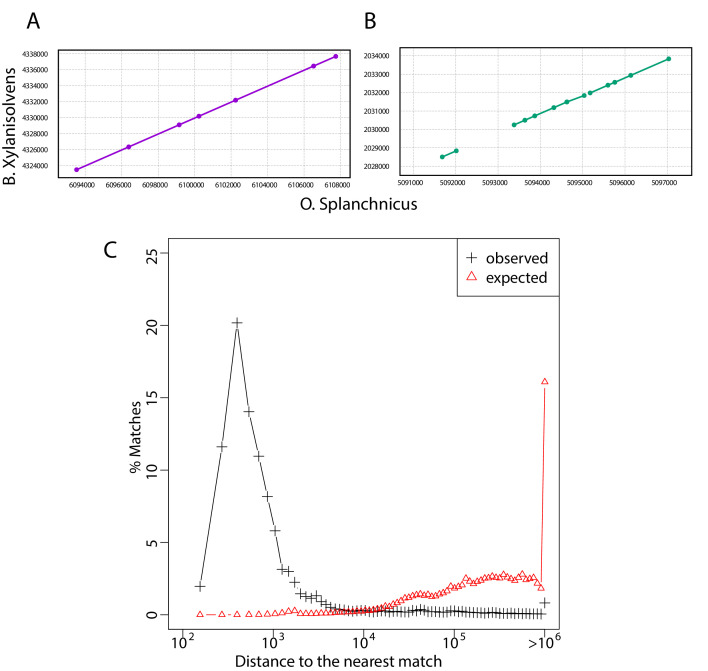
Long exact matches cluster in bacterial genomes. (**A-B**) Dot plots resulting from the comparison of two regions of *Bacteroides xylanisolvens* (strain_H207) and *Odoribacter splanchnicus* (DSM_20712). Lines represent exact matches longer than 200 bp and large dots represent the end of such exact matches, to highlight short mismatches in between long exact matches. (**C**) Histogram of the distance to the nearest match for each match of the curated dataset. For each comparison with at least two exact matches >300 bp, we compute, in each species for each match, its distance to the nearest match found in the same pairwise comparison. Black plus signs present the distribution of distances to the nearest match. If matches were uniformly distributed over genomes, the distance between a match and its nearest counterpart in the comparison of species a and b should be of the order of $D_{a}=L_{a}/N_{a\,b}$, where $L_{a}$ is the length of genome of species a and $N_{a\,b}$ the number of exact matches between species a and b. Red plus signs present the distribution of $D_{a}$ for all comparisons with at least two matches. The large difference between the two distributions shows that matches are not uniformly distributed over genomes. See Restricted dataset for a description of the restricted dataset.

**Figure 1—figure supplement 2.**
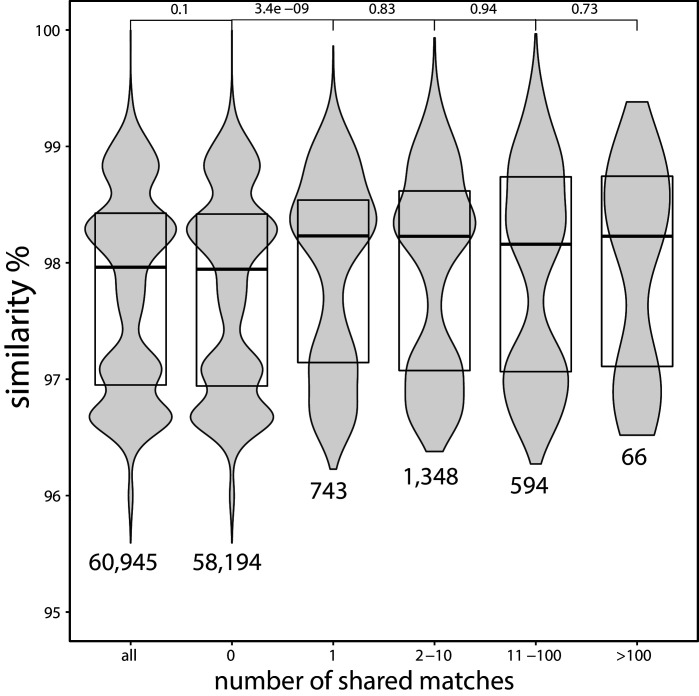
Average nucleotide identity (ANI) between strains of *Escherichia coli* which share a certain number of exact matches to a different family. ANI (obtained from Rodriguez-R et al., 2018) between strains of *E. coli* which share a certain number (horizontal axis) of exact matches to a different family, based on the matches found in the curated set (strains without matches to genomes in a different family are filtered out in this analysis). The p-values on the top are obtained using the Wilcoxon rank-sum test. The numbers below the distributions indicate the total number of pairs with the corresponding number of shared matches. This figure suggests that strains which share matches to distant species tend to be more similar genetically.

**Figure 1—figure supplement 3.**
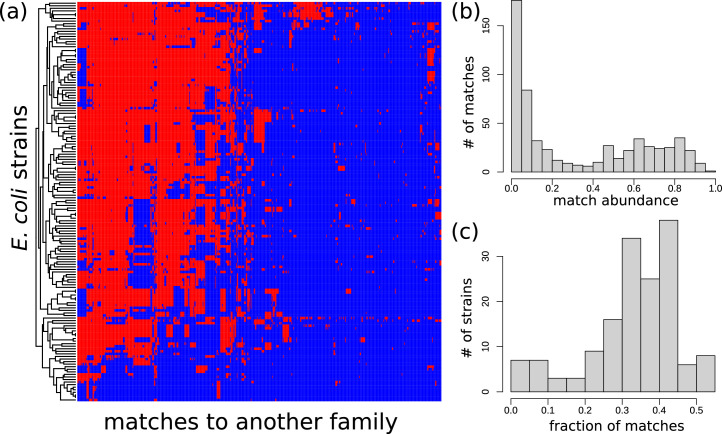
Distribution of exact matches between *Escherichia coli* strains and bacteria from a different family. Distribution of exact matches between *E. coli* strains and bacteria from a different family. (See Section Appendix 1 Phylogenetic analysis among HGT event in *E. coli*.) (**a**) For all long matches between *E. coli* and genomes from a different family found in the curated dataset, we determined whether a blastn hit with E-value $<10^{-5}$ is present in 156 strains of *E. coli*. The heatmap visualises the results: blue represents absence, red represents presence. blastn found 32,446 hits, but only 598 matches with a unique presence/absence pattern are displayed in this figure (i.e., filtered matches have exactly the same presence/absence pattern in the strains as one of the matches in the figure). Rows (strains) and columns (matches) were hierarchically clustered. (**b**) For the set of matches of panel (**a**), this panel presents a histogram of the proportion of *E. coli* strains that possess these matches. A bimodal distribution is apparent. (**c**) For the set of *E. coli* strains of panel (**a**), this panel shows a histogram of the fraction of the matches that they posses.

**Figure 1—figure supplement 4.**
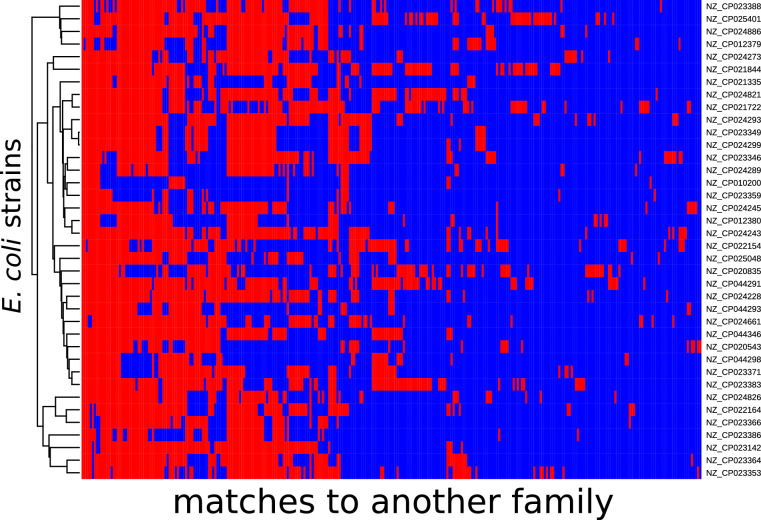
Distribution of exact matches between *Escherichia coli* strains and bacteria from a different family. Distribution of exact matches between *E. coli* strains and bacteria from a different family. The same as Figure 1—figure supplement 3, but for 35 *E. coli* strains with average nucleotide identity (ANI) distances taken from Rodriguez-R et al., 2018. The dendrogram represents the UPMGA tree based on the ANI distances.

Because very long exact sequence matches are extremely unlikely in orthologs, those that do occur are most likely xenologs: sequences that are shared due to relatively recent events of HGT. As an example, Figure 1C shows a dot plot comparable to Figure 1A, but now comparing the genomes of *Enterococcus faecium* and *Atopobium minulum*. No diagonal line is seen because these genomes belong to different phyla and therefore have low sequence identity. Nevertheless, an exact match spanning 19,117 bp is found (diagonal green line highlighted by the red ellipse). The most parsimonious explanation for such a long match is a recent HGT event. In addition, the GC content of the match (55%) deviates strongly from that of both contigs (38.3% and 48.9%, respectively), another indication that this sequence originates from HGT (Ravenhall et al., 2015). Comparing the sequence of this exact match with all non-redundant GenBank CDS translations using blastx (Altschul et al., 1990), we find very strong hits to VanB-type vancomycin resistance histidine, antirestriction protein (ArdA endonuclease), and an LtrC-family phage protein that is found in a large group of phages that infect Gram-positive bacteria (Quiles-Puchalt et al., 2013). Together, this suggests that the sequence was transferred by transduction and established in both bacteria aided by natural selection acting on the conferred vancomycin resistance.

In the following, we assume that long identical DNA segments found in pairs of bacteria belonging to different genera reveal HGT. This assumption is further supported by several observations. First, in the identified matches, we did not detect enrichment of sequences known to be highly conserved, such as rRNA (see functional analysis below in HGT rates of genes differ strongly between functional categories). Second, the exact matches are clustered in the genomes (see Figure 1—figure supplement 1 (C)), as expected for transferred sequence that have already started to diverge in the two species, giving rise to several shorter adjacent matches. Third, if it is true that long exact matches are the result of HGT events, closely related strains should present similar long exact matches to distant species, resulting from HGT event that occurred prior to the split of the two strains. We do observe such a pattern (see Figure 1—figure supplement 2, Figure 1—figure supplement 3 and Figure 1—figure supplement 4) although the signal is not very strong (see Appendix 1 Phylogenetic analysis among HGT event in *E. coli* for more details). We stress, however, that a matching sequence may not have been transferred directly between the pair of lineages in which it was identified: more likely, it arrived in one or both lineages independently, for instance carried by a phage or another mobile genetic element that transferred the same genetic material to multiple lineages through independent interactions.

We restrict this study to matches longer than 300 bp to minimise the chance that those matches result from vertical inheritance. Because after HGT the transferred sequences accumulate mutations, matches longer than 300 bp are expected to originate from relatively recent events. Assuming a generation time of 10 hr (Gibson et al., 2018), we estimate the detection horizon to be of the order of 1000 years ago (see Age-range estimation of the exact matches in Materials and methods).

### Empirical length distributions of exact matches obey a power law

To study HGT events found in pairs of genomes, we considered the statistical properties of r, the length of exact matches. Note that the number of long matches found in a single pair of genomes is usually very small. Hence, in this study we conduct all statistical analyses at the level of genera. To do so, we selected all bacterial genome fragments longer than 10^5^ bp from the NCBI RefSeq database (1,343,042 in total) and identified all sequence matches in all pairs of sequences belonging to different genera ($\approx 10^{9}$ pairs). We then analysed the distribution of the lengths of the matches, called the match-length distribution or MLD. The MLD for a pair of genera $G_{A}$ and $G_{B}$ is defined as the normalised length distribution of the matches found in all pairwise comparisons of a contig from $G_{A}$ and a contig from $G_{B}$. The normalisation ensures that the prefactor of the MLD does not scale with the number of genomes present in the database (see Empirical calculation of the MLD for pairs of genera and sets of genera in Materials and methods). A comparable approach has previously been applied successfully to analyse the evolution of eukaryotic genomes (Gao and Miller, 2011; Massip and Arndt, 2013; Massip et al., 2015).

**Figure 2.**
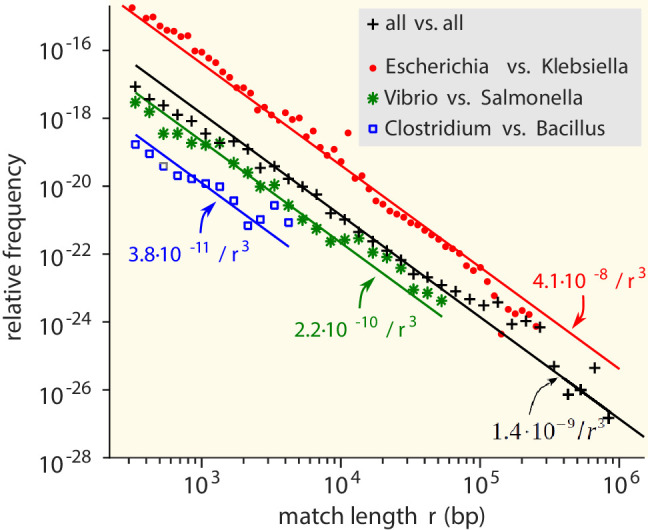
Match-length distributions (MLDs) obtained by identifying exact sequence matches in pairs of genomes from different genera, based on matches between *Escherichia* and *Klebsiella* (red dots), *Vibrio* and *Salmonella* (green stars), and *Clostridium* and *Bacillus* (blue squares). Black plus signs represent the MLD obtained by combining the MLDs for *all* pairs of genera. Each MLD is normalised to account for differences in the number of available genomes in each genus (see Empirical calculation of the MLD for pairs of genera and sets of genera in Materials and methods). Only the tails of the distributions (length r≥300) are shown. Solid lines are fits of power laws with exponent −3 (Equation (1)) with just a single free parameter. Figure 2—source data 1.MLD obtained by combining the MLDs for *all* pairs of genera. Figure 2—source data 2.MLD based on matches between *Clostridium* and *Bacillus.* Figure 2—source data 3.MLD based on matches between *Vibrio* and *Salmonella.* Figure 2—source data 4.MLD based on matches between *Escherichia* and *Klebsiella.*

We first consider the MLD obtained by combining the MLDs for all pairs of genera. While the vast majority of matches is very short (<25 bp), matches with a length of at least 300 bp do occur and contribute a fat tail to the MLD (Figure 2). Strikingly, over many decades this tail is well described by a power law with exponent −3: 

(1) $$m\,\left(r\right)∼r^{-3}.$$

**Figure 2—figure supplement 1.**
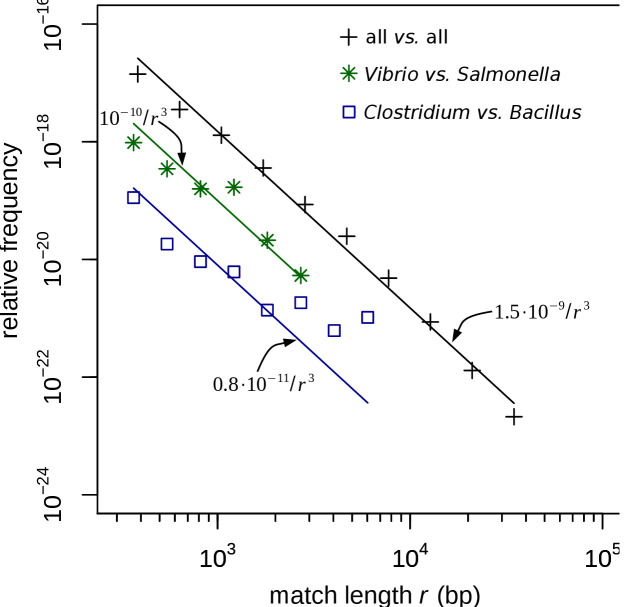
Match-length distributions (MLDs) obtained by identifying exact sequence matches in pairs of genomes from different genera in the curated set. MLDs obtained by identifying exact sequence matches in pairs of genomes from different genera. In contrast to Figure 2, for this figure the restricted dataset was used, based on contigs larger than 10^6^ bp that were manually curated, as described in Restricted dataset in Materials and methods. MLDs are shown based on matches between *Vibrio* and *Salmonella* (green stars), and *Clostridium* and *Bacillus* (blue squares). Black plus signs represent the MLD obtained by combining the MLDs for all pairs of genera *from different families*. (Remember that the restricted dataset only contains matches between contigs belonging to different families.) Each MLD is normalised to account for differences in the number of available genomes in each genus (see Empirical calculation of the MLD for pairs of genera and sets of genera in Materials and methods). Only the tails of the distributions (length r≥300) are shown. Solid lines are fits of power laws with exponent −3 (Equation (1)) with just a single free parameter.

**Figure 2—figure supplement 2.**
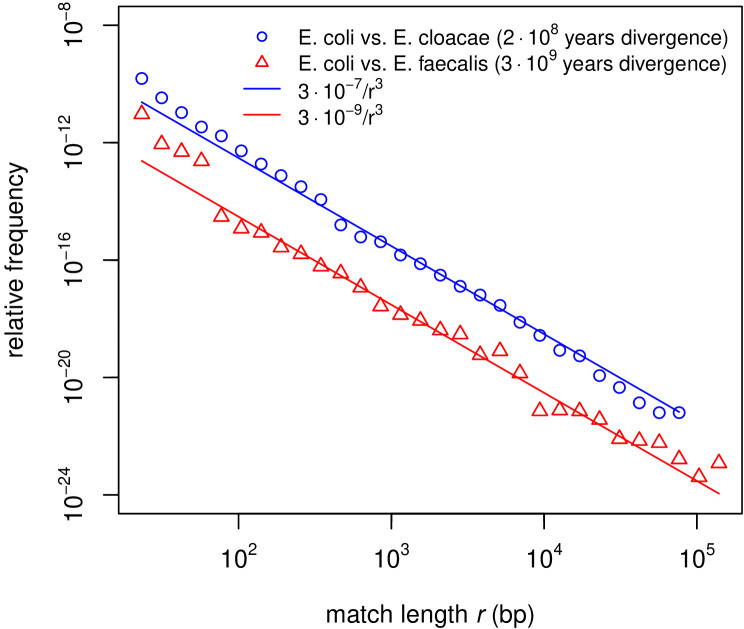
Match-length distributions (MLDs) computed for shorter matches. MLDs obtained by identifying exact sequence matches in pairs of genomes from different species (see legend). Each MLD is normalised to account for differences in the number of available genomes in each genus (see Empirical calculation of the MLD for pairs of genera and sets of genera in Materials and methods). Here, shorter matches (r≥20) are shown. Solid lines are fits of power laws with exponent −3 (Equation (1)) with just a single free parameter (see legend). For short matches (r<100 bp), the MLDs deviate from the power law with exponent −3.

The same −3 power law is found in the MLDs for individual pairs of genera (see Figure 2).

To verify that the observed power-law distributions were not the result of assembly artefacts or erroneous annotation, we constructed a smaller, manually curated dataset which included only long contigs ($>10^{6}$ bp, see Restricted dataset in Materials and methods). This *restricted* dataset still comprises 138,273 matches longer than 300 bp. MLDs computed with this dataset also consistently results in −3 power laws ( Figure 2—figure supplement 1). Hence, the results are robust to assembly or annotation artefacts.

Below, we will explain that the power law is a signature of HGT. Consistently, for matches shorter than 300 bp, the MLDs deviate from the power law (see Figure 2—figure supplement 2), because in this regime vertical inheritance, convergent evolution and random matches contribute to the MLD.

### A simple model of HGT explains the power-law distribution of exact sequence matches

A simple model based on a minimal set of assumptions can account for the power law observed in the MLD (see Box 1). Let us assume that, due to HGT, a given pair of bacterial genera A and B obtains new long exact matches at a rate ρ, and that these new matches have a typical length K much larger than 1 bp. These matches are established in certain fractions $f_{A}$ and $f_{B}$ of the populations of the genera, possibly aided by natural selection. Subsequently, each match is continuously broken into shorter ones due to random mutations that happen at a rate μ per base pair in each genome. Then the length distribution of the broken, shorter matches, resulting from all past HGT events, converges to a steady state that for 1≪r<K is given by the power law $m(r)=A/r^{3}$, with prefactor:

(2) $$A:=K\,\frac{f_{A}\,f_{B}}{L_{A}\,L_{B}}\,\frac{\rho }{\mu },$$

consistent with Equation (1); see Analytical calculation of the MLD predicted by a simple model of HGT in Materials and methods for a full derivation. Here $L_{A}$ (resp. $L_{B}$) is the average genome length of all species in genus A (resp. B). Hence, the power-law distribution can be explained as the combined effect of many HGT events that occurred at different times in the past. While the model above makes several strongly simplifying assumptions, many of these can be relaxed without affecting the power-law behaviour; see Robustness of the power-law behaviour in Materials and methods for an extended discussion.

Box 1. Horizontal gene transfer explains the power-law distribution of exact sequence matches.

The tails of the match-length distributions (MLDs) in Figure 2 obey a power-law distribution with exponent −3. This observation can be explained by a simple model of horizontal gene transfer (HGT). (See A simple model of HGT explains the power-law distribution of exact sequence matches and Analytical calculation of the MLD predicted by a simple model of HGT for a full derivation.) Consider two genomes, A and B, from different genera (see Box 1—figure 1, left panel). At some point in time, HGT introduces a new, long exact match between the two genomes (coloured bar). Subsequently, mutations (red stars) have the effect of ‘breaking’ this match into ever smaller pieces (see Figure 1—figure supplement 1A-B for two examples). With time, more and more mutations accumulate. The more time passes, the more pieces there will be, but the shorter they will be on average. Assuming that mutations occur at random positions, after some time the lengths of the exact matches within this one segment are distributed exponentially (bottom left). With time, the mean of this exponential distribution decreases. Each MLD in Figure 2 represents a collection of exact matches obtained by comparing many pairs of genomes and thus contains contributions of many xenologous segments created at various times in the past. Therefore, these distributions are the result of mixtures of many exponential MLDs, each with a different mean. Mathematically, such a mixture becomes a power law with exponent −3 provided the age of the xenologous segments is not strongly biased. Box 1—figure 1 illustrates this point (right panel). If 50 exponential MLDs (grey, blue, and purple curves) based on randomly sampled ages are simply summed up, the result (red curve) approaches a power law with exponent −3, recognised in a log-log plot as a straight line with a slope of −3.

**Box 1—figure 1.**
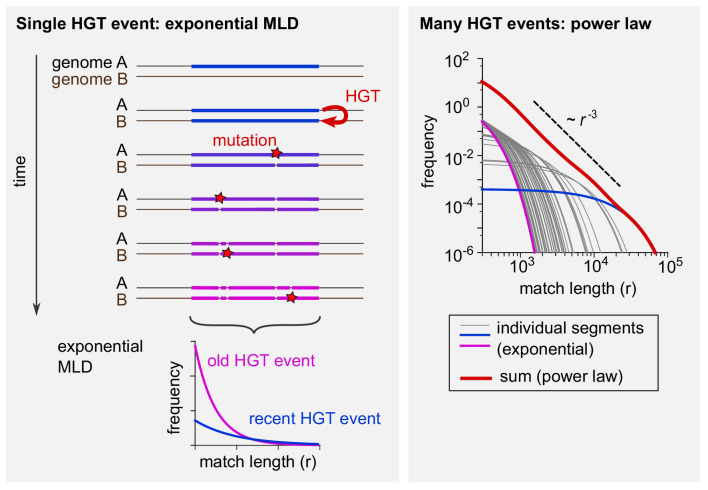
Schematic explanation of the mathematical model. (Left) The evolutionary fate of a DNA segment following HGT. Initially, the event generates a single long exact match between genomes A and B. As time passes, mutations break this match into more and more pieces that are shorter and shorter. The MLD stemming from a single segment follows an exponential distribution with a mean decreasing with the age of the transfer, as represented at the bottom of the scheme. (Right) Exponential MLDs (log-log scale) for many segments originating from different HGT events (blue: very recent event, purple: older event). The red curve is the sum of all blue, purple and grey curves and follows a power law with exponent –﻿3.

In the model, the prefactor A quantifies the abundance of long exact matches and hence is a measure of the rate with which two taxa exchange genetic material. Equation (2) shows that A reflects the bare rate of the transfer events, the typical length of the transferred sequences, as well as the extent to which the transferred sequences are established in the receiving population, possibly aided by selection. By contrast, because of the normalisation of the MLD (see Empirical calculation of the MLD for pairs of genera and sets of genera in Materials and methods), A does not scale with the number of genomes in the genera being compared and is thus robust to sampling noise. Hence, the value of A can be used to study the variation in HGT rate among genera. In addition, the values of A estimated from the full and the restricted datasets (Figure 2 and Figure 2—figure supplement 1) are very close, showing that the estimates of A are robust to assembly artefacts. Finally, our estimates are unlikely to be strongly affected by the presence of plasmids since only a small fraction of plasmids is longer than 10^5^ or 10^6^ bp (Shintani et al., 2015).

### Long-distance gene exchange is widespread in the bacterial domain

The analysis above has allowed us to identify a large number of HGT events. In addition, the derivations in the previous section provide a method to quantify the effective HGT rate between any two taxa by measuring the prefactor A. Supplementary file 1 and 2 contains the value of A for all pairs of genera and families. Using these results, we further studied the HGT rate between all pairs of bacterial families in detail.

**Figure 3.**
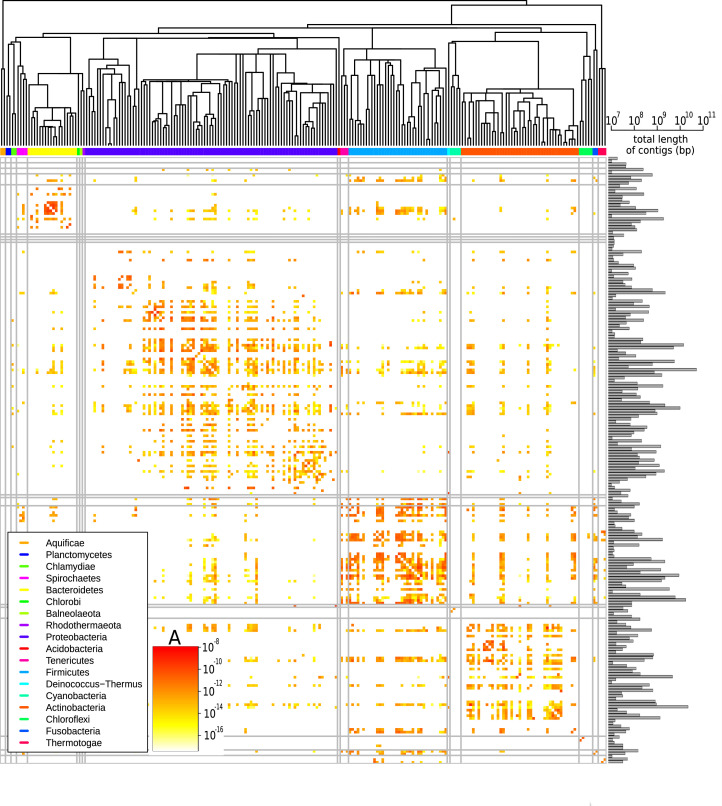
Effective pairwise horizontal gene transfer (HGT) rate at the family level. For each pair of families, the prefactor A is displayed (decimal logarithmic scale, see colourbar and Supplementary file 1). The values on the diagonal are set to zero. The phylogenetic tree of bacterial families, taken from Kumar et al., 2017, is shown at the top. Phyla are indicated with coloured bars next to the upper axes of the heatmap (see legend); grey vertical and horizontal lines represent borders between phyla. The barplot on the right-hand side of the heatmap shows the cumulative genome sizes of each family (decimal logarithmic scale).

Figure 3 shows the prefactors A for all pair of families (see Figure 3—figure supplement 1 for a similar plot for all pairs of phyla). Families for which the available sequence data totals less than 10^7^ bp were filtered out since in such scarce datasets typically no HGT is detected (Figure 3—figure supplement 2) and the prefactor cannot reliably be estimated (see Supplementary file 3 for the total length of all families). A first visual inspection of the heatmap reveals that the HGT rate varies drastically (A varies from $10^{-16}$ to $10^{-8}$) among pairs of families. Also, the large squares on the diagonal of the heatmap indicate that HGT occurs more frequently between taxonomically closely related families. This is especially apparent for well-represented phyla including Bacteroidetes, Proteobacteria, Firmicutes, and Actinobacteria. Yet, we also observe high transfer rates between many families belonging to distant phyla, indicating that transfer events across phyla are also frequent (see Figure 3—figure supplement 1). Notably, we find that some families display an elevated HGT rate with all other families across the phylogeny; these families are visible in the heatmap (Figure 3) as long colourful lines, both vertical and horizontal.

**Figure 3—figure supplement 1.**
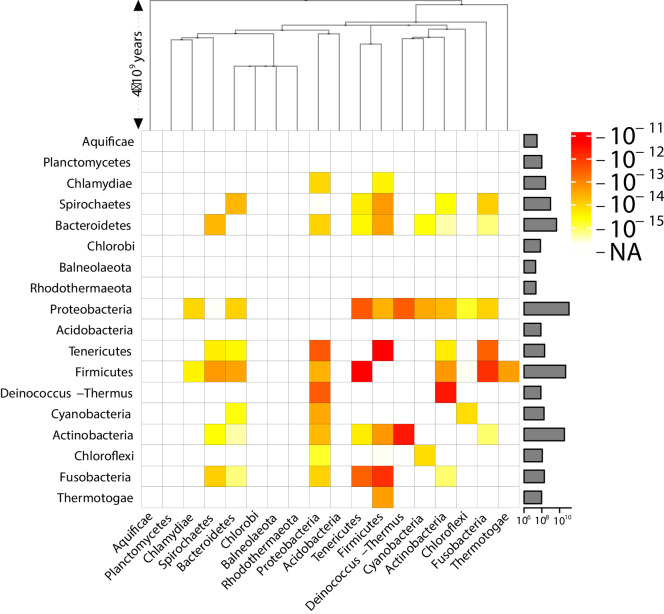
Effective pairwise horizontal gene transfer (HGT) rate at the phylum level (coarse-grained version of Figure 3). For each pair of phyla, the prefactor A is displayed (decimal logarithmic scale, see colourbar). On the diagonal, the values are set to NA. The barplot on the right-hand side of the heatmap shows the total length of the available contigs for each phylum (decimal logarithmic scale).

**Figure 3—figure supplement 2.**
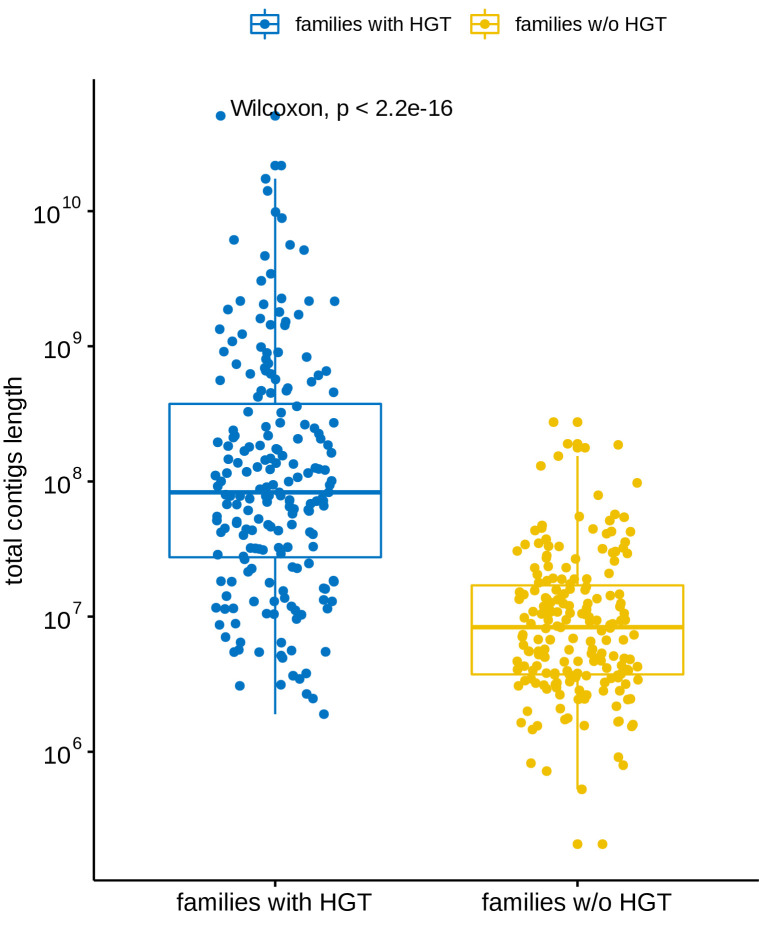
Total contig length distribution of families versus their involvement in long-distance horizontal gene transfer (HGT) events. Blue: Families involved in at least one HGT event with another family. Yellow: Families not involved in any of the observed long-distance HGT event. The total contig length of a family is defined as the sum of the length of all the contigs belonging to that family.

**Figure 4.**
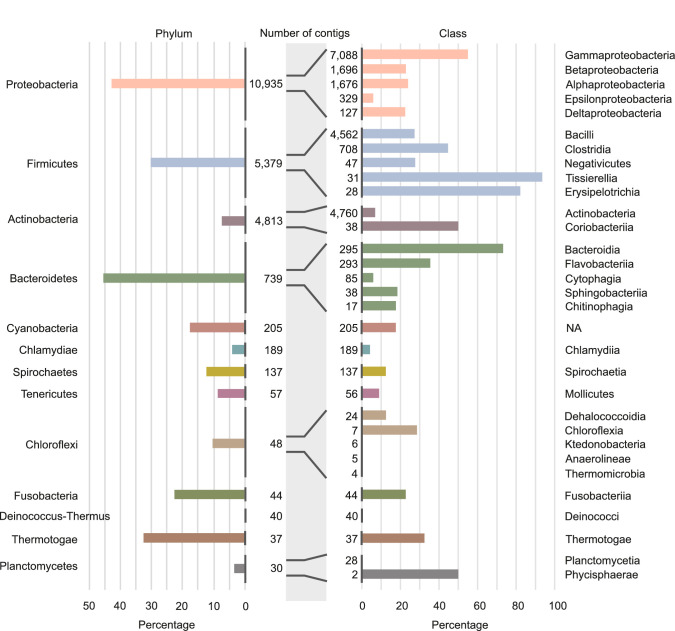
Involvement of different phyla and classes of bacteria in long-distance horizontal gene transfer (HGT). Percentage of contigs involved in at least one of the observed long-distance HGT event grouped at phylum level (left panel) and at classes level (right panel). Note that only the classes with the largest numbers of contigs are shown (see Supplementary file 4 for all data). Numbers of contigs belonging to the phyla and classes are given in the middle part of figure.

We studied the HGT rate variations in more detail in the restricted dataset (see Restricted dataset in Materials and methods). The analysis of the restricted dataset reveals the extent of HGT, even between distant species (Figure 4). Indeed, we find that 32.6% of RefSeq species have exchanged genetic material with a species from a different family. Moreover, we find that 8% of species in the database have exchanged genetic material with a species from a different phylum. Finally, the species involved in these distant exchanges are spread across the phylogenetic tree: the species involved in long-distance transfers belong to 19 different phyla (out of 34). Importantly, we repeat that the method is sensitive only to events that occurred in the last ∼1000 years. Also, these estimates are lower bound estimates since the power of our detection method is limited in species for which only few genomes have been sequenced.

The data also unveil that the propensity to exchange genetic material varies dramatically among species from closely related classes. For instance, within the phylum Firmicutes, we find classes in which we detected HGT in only a small percentage of species (30% in the Negativicutes), while in other classes we find events in almost all species (>90% in Tissierellia, Figure 4 and Supplementary file 4). This trend can be observed in most of the phyla and raises the question of which species features drive HGT rate variations.

### The rate of HGT correlates with evolutionary distance, ecological environment, Gram staining, and GC content

To better understand the causes of the large variations in transfer rate between different taxa, we next studied the effect of biological and environmental properties on the HGT rate.

**Figure 5.**
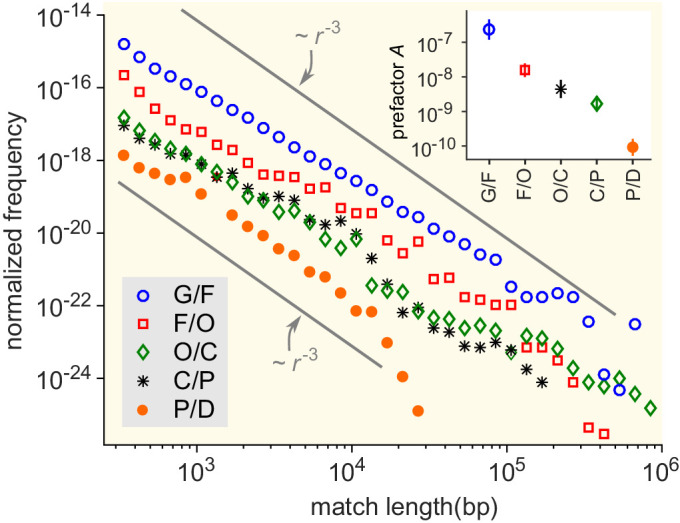
Match-length distributions (MLDs) resulting from comparison of genera at a given taxonomic distance. Statistically, the prefactor A obtained for a pair of genera decreases with the taxonomic distance between those genera. To demonstrate this, the figure shows averaged MLDs based on the MLDs of all pairs of genera at given taxonomic distances. G/F (blue circles): MLD obtained by averaging MLDs of pairs of genera that belong to the same family. F/O (red squares): MLD obtained by averaging MLDs of pairs of genera that belong to the same order, but to different families. O/C (green diamonds): Pairs of genera from the same class, but different orders. C/P (black stars): Same phylum, different classes. P/D (red circles): Same domain, different phyla. Grey lines indicate power laws $m\,\left(r\right)\propto r^{-3}$, for comparison. Inset: Prefactor A for each of the distributions in the main figure. The prefactor decreases by orders of magnitude as the taxonomic distance increases. Figure 5—source data 1.Raw data corresponding to the inset of Figure 5: prefactor A for each of the distributions in the main figure. Figure 5—source data 2.MLD obtained by averaging MLDs of pairs of genera that belong to the same domain, but to different phyla (P/D). Figure 5—source data 3.MLD obtained by averaging MLDs of pairs of genera that belong to the same phylum, but to different classes (P/C). Figure 5—source data 4.MLD obtained by averaging MLDs of pairs of genera that belong to the same class, but to different orders (O/C). Figure 5—source data 5.MLD obtained by averaging MLDs of pairs of genera that belong to the same order, but to different families (F/O). Figure 5—source data 6.MLD obtained by averaging MLDs of pairs of genera that belong to the same family (G/F).

First, we assessed the impact of the taxonomic distance between genera. To do so, we computed the prefactor A for pairs of genera at various taxonomical distances (Figure 5). On average, this prefactor decreases by orders of magnitude as the taxonomic distance between the genera increases (inset of Figure 5). In particular, the average prefactor obtained when considering genera from the same family is more than three orders of magnitude higher than when considering genera from different phyla. Seeking exact matches between organisms from different domains, we compared genomes of bacteria and archaea and found only a few long matches (see "Comparing bacterial and archaeal genomes" section in the Appendix). These results support the notion that the divergence between organisms plays an important role in the rate of HGT between them (Ochman et al., 2000; Brügger et al., 2002; Nakamura et al., 2004; Ge et al., 2005; Choi and Kim, 2007; Dagan et al., 2008; Andam and Gogarten, 2011) (see also Figure 5—figure supplement 1). Note, however, that a lower effective rate of HGT can be due to a lower transfer rate of genetic material and/or a more limited fixation in the receiving genome, and the model cannot distinguish those two scenarios.

**Figure 5—figure supplement 1.**
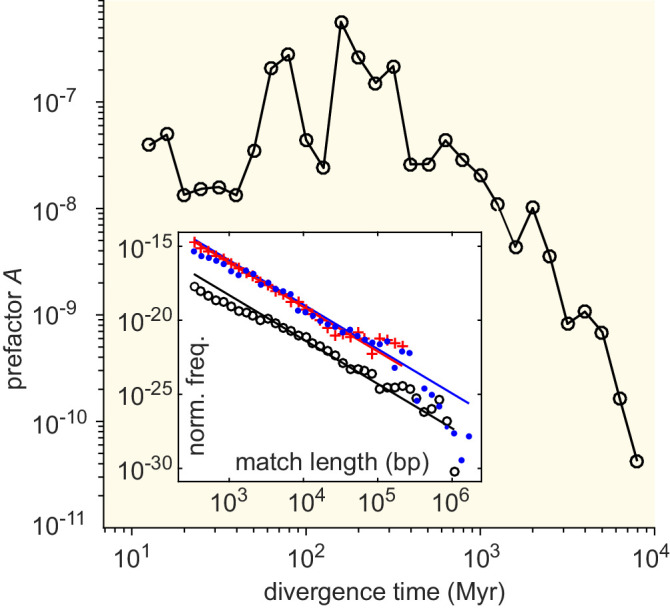
Effective horizontal gene transfer (HGT) rate as a function of the divergence time between genera. Divergence times (in Myr) were obtained from Kumar et al., 2017. Main panel: The prefactor A of the MLD resulting from the comparison of pairs of genera with a given divergence time (binned). Inset: MLD for divergence times in the intervals $10^{1}-10^{2}$ Myr (blue dots), $10^{2}-10^{3}$ Myr (red plus signs), and $10^{3}-10^{4}$ Myr (black circles). Lines represent $r^{-3}$ dependence.

**Figure 5—figure supplement 2.**
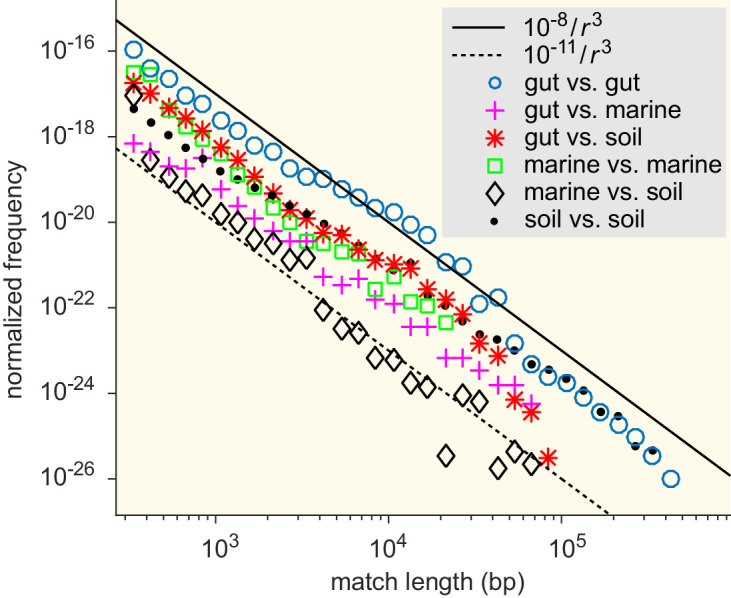
Match-length distributions (MLDs) resulting from comparison of sets of genera associated with different ecological environments: gut, soil, and marine (see Supplementary file 7 for detailed annotation).

**Figure 5—figure supplement 3.**
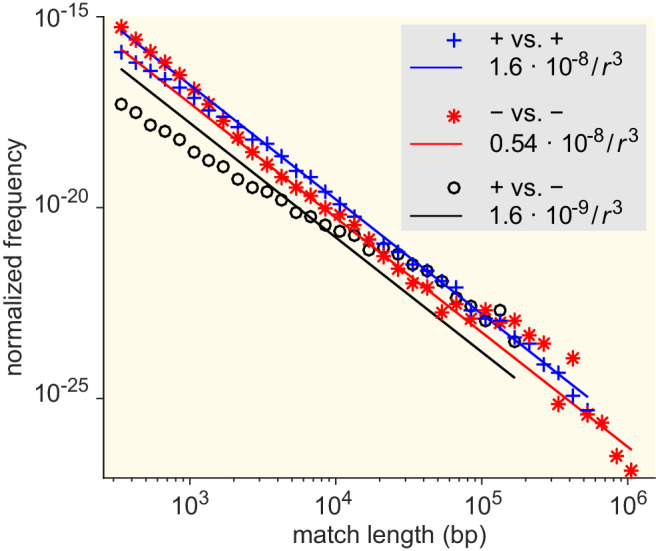
Match-length distributions (MLDs) resulting from comparison of sets of genera associated with different Gram staining test results (see Supplementary file 7 for detailed annotation).

**Figure 5—figure supplement 4.**
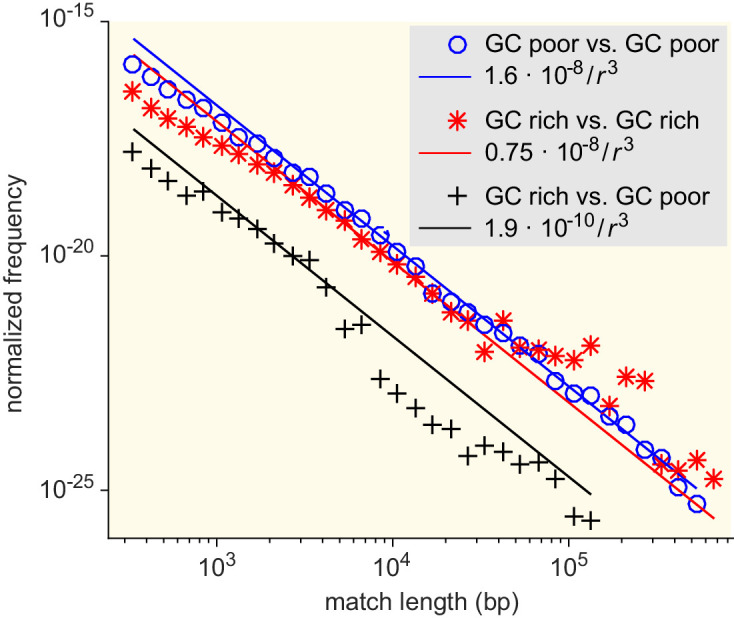
Match-length distributions (MLDs) resulting from comparison of sets of bacteria associated with different GC content (see Supplementary file 7 for detailed annotation).

We then explored other factors that influence the value of A. To do so, we calculated MLDs for sets of genera from different ecological environments: gut, soil, or marine (Figure 5—figure supplement 2), regardless of their taxonomic distance. Our results suggest that the effective rate of HGT is about 1000 times higher among gut bacteria than among marine bacteria. This pattern is observed for both the rates of HGT within ecological environments (i.e., HGT among gut bacteria versus among marine bacteria) and the rates of crossing ecological environments (i.e., HGT between gut and soil bacteria versus between marine and soil bacteria). The soil bacteria take an intermediate position between the gut and the marine bacteria. Moreover, bacteria from the same environment tend to share more matches than bacteria from different environments, consistent with previous analyses (Smillie et al., 2011).

A similar analysis demonstrates that the HGT rate among Gram-positive bacteria and among Gram-negative bacteria is much larger than between these groups (see Figure 5—figure supplement 3). The groups of bacteria with GC-poor and GC-rich genomes exhibit a similar pattern (see Figure 5—figure supplement 4). We note, however, that all these factors correlate with each other (Gupta, 2000). From our analysis, the contribution of each factor to the effective rate of HGT therefore remains unclear.

### HGT rates of genes differ strongly between functional categories

**Figure 6.**
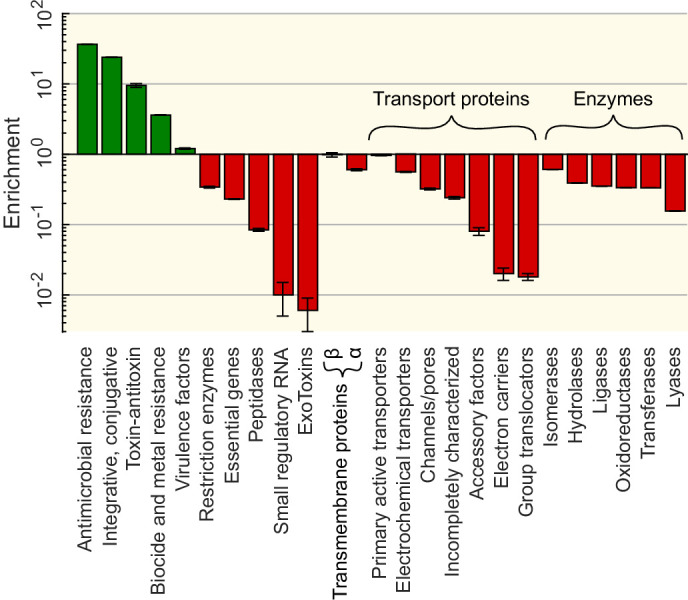
Functional enrichment of the sequences involved in horizontal gene transfer (HGT) based on the analysis of 12 specialised databases. Enrichments for each gene category (vertical axis) are computed relative to a control set obtained by sampling random sequences from the contigs that contained the matches (see Materials and methods). Enrichment for genes offering resistance against various types of antibiotics and biocides can be found in Supplementary file 5.

To better understand the factors that explain variations in observed HGT rates, we next conducted a functional analysis of transferred sequences. To determine whether particular functions are overrepresented in the transferred sequences, we first queried 12 databases, each specifically dedicated to genes associated with a particular function. Comparing to a randomised set of sequences (see Gene enrichment analyses in Materials and methods) reveals that the gene functions of the transferred sequences strongly impact the transfer rate, as we observe a 3.5 orders of magnitude variation between the most and the least transferred categories (Figure 6 and Appendix 1—table 1).

More specifically, antibiotic and metal resistance genes are among the most widely transferred classes of genes (resp. 37× and 4× enrichment compared to random expectation), in good agreement with previous evidence (Huddleston, 2014; von Wintersdorff et al., 2016; Evans et al., 2020). The enrichment of resistance genes is expected since their functions are strongly beneficial for bacterial populations under specific, transient conditions. Interestingly, genes providing resistance against tetracycline and sulfonamide antibiotics – the oldest groups of antibiotics in use – are the most enriched (see the full list in Supplementary file 5). In addition, we also find a strong enrichment among the transferred genes of genes classified as integrative and conjugative elements, suggesting that these genes mediated the HGT events (Paquola et al., 2018; Nakamura et al., 2004). In contrast, exotoxins and small regulatory RNAs are the least transferred genes (≈100× depletion). More generally, genes in the wider ‘Transport proteins’ and ‘Enzymes’ categories are strongly underrepresented in the detected HGT events.

To obtain a better understanding of the function of the transferred sequences, we annotated the transferred sequences in two ways: using SEED subsystems (Overbeek et al., 2005) and using gene ontology (GO) terms (Ashburner et al., 2000; Carbon et al., 2021). While the 12 curated databases queried above are more complete and accurate on their specific domains, using the SEED subsystem and GO analysis allows to test for over- or underrepresentation in a systematic way and for a broader set of functions. To avoid false positives, we retained only the functions for which the results showed good agreement between the SEED and GO approaches (see SEED subsystems and GO terms ontological classification in Materials and methods).

The results of these functional profiling methods are in good agreement with the database queries as the broad categories linked to ‘Phages, Prophages, Transposable elements, Plasmids’, and to ‘Virulence, Disease, and Defense’ are found to be the most enriched, although with a smaller enrichment (4.6- and 3.4-fold enrichment, respectively, see Supplementary file 6). Those findings confirm the strong enrichment of expected functional categories among HGT events and validate the good resolution of our methods.

In addition to previously known enriched functions, we also discovered a significant enrichment (1.3×, conditional test adjusted p-value <10^−16^, see SEED subsystems and GO terms ontological classification in Materials and methods) for genes in the ‘iron metabolism’ class. Indeed, a wide range of iron transporters, parts of siderophore, and enzymes of its biosynthesis appeared in our HGT database, in line with previous analysis focusing on cheese microbial communities (Bonham et al., 2017). Hence, the results show that the horizontal transfer of genes related to iron metabolism occurs in a wide set of species and is not restricted to species found in cheese microbial communities. Notably, the proteins in the ‘iron metabolism’ functional category can be identified in transferred sequences belonging to six different bacterial phyla.

Two other interesting SEED categories are found to be enriched. First, we found an enrichment for genes belonging to the secretion system type IV (1.4× enrichment, conditional test adjusted p-value <10^−14^). Among the seven types of secretion systems (Costa et al., 2015), we found an enrichment only for type IV, a diverse and versatile secretion system, which has been shown to play a role in the prokaryotic conjugation process (Wallden et al., 2010). Finally, we also found an enrichment for proteins involved in spore DNA protection, an important step of the complex sporulation process (Piggot and Hilbert, 2004). This enrichment is in good agreement with the recent finding that some bacteria rely on HGT to acquire sporulation genes, although the mechanisms and benefits of this strategy are still unclear (Ramos-Silva et al., 2019).

## Discussion

In this study, we developed a computationally efficient method to identify recent HGT events. Applying this method to the genomes of the 93,481 organisms of the RefSeq database, we identified an unprecedentedly large number of HGT events between bacterial genera. Our analysis reveals that HGT between distant species is extremely common in the bacterial world, with 32.6% of organisms detected as having taken part in an event that crossed genus boundaries in the last ∼1000 years. While a similar analysis has been conducted on a much smaller dataset (about 2300 organisms, see Smillie et al., 2011), this study is, to our knowledge, the first to provide an extensive description of HGT in the microbial world at this scale.

One striking result is the finding that HGT is also common between very distant organisms. Indeed, in 8% of the organisms we studied, we found evidence for their involvement in a transfer of genetic material with bacteria from another phylum in the last ∼1000 years. The molecular mechanisms at play in these long-distance transfer events remain to be elucidated, for instance via a dedicated study targeting families with very high exchange rate.

Analysing the statistical properties of the exact sequence matches between different genera, we found that the tail of the MLD follows a power law with exponent −3. This observation is particularly robust since the empirical power law spans between two and four orders of magnitude, with an exponent always close to −3. To understand this phenomenon, we developed a model of HGT that explains the observed −3 power law. The prefactor of the power law depends on a single lumped parameter: the effective HGT rate. This made it possible to quantify the effective rate of HGT between any pair of genera. Doing so, we found that the HGT rate varies dramatically between pairs of taxa (Figure 3 and Figure 3—figure supplement 1), raising the question of which factors influence the HGT rate. Further analysis confirmed that the HGT rate decreases with the divergence between the two bacteria exchanging material (Figure 5 and Figure 5—figure supplement 1) and is larger for pairs of bacteria with similar properties, such as ecological environment, GC content, and Gram staining (Figure 5—figure supplement 2, Figure 5—figure supplement 3 and Figure 5—figure supplement 4). However, since all these properties are correlated, we could not disentangle the independent contribution of each of those features to the HGT rate.

Finally, a functional analysis of the transferred sequences showed that the function of a gene also strongly affects its chance of being exchanged (Figure 6). As expected, genes conferring antibiotic resistance are the most frequently transferred. In contrast, some functional categories are strongly underrepresented in the pool of transferred genes. For instance, genes that are involved in transcription, translation, and related processes as well as those involved in metabolism are all depleted in our HGT database. One potential explanation is that these genes generally co-evolve with their binding partners (Jain et al., 1999). As such, their transfer would be beneficial to the host species only if both the effector and its binding partner were to be transferred together. As simultaneous HGT of several genes from different genome loci is very unlikely (unless they are co-localised), these genes are less prone to HGT. For additional discussion of the functional constraints on HGT, we refer to these reviews (Thomas and Nielsen, 2005; Popa and Dagan, 2011; Pál et al., 2005; Cohen et al., 2011).

Our model of HGT is very robust to its simplifying assumptions. Most of them can be relaxed without breaking the specific power-law behaviour with the −3 exponent. In fact, the only crucial assumptions of the model are that HGT events have taken place continuously and at a non-zero rate up to the present time (see Materials and methods). Whether HGT is a continuous process on evolutionary time scales or instead occurs in bursts has been a matter of debate (Rivera et al., 1998; Jain et al., 1999; Wolf and Koonin, 2013), and bursts of transfer events at some point in the past might explain some of the deviations from the −3 power-law behaviour we observe (Figure 5). In addition to HGT bursts, other complex evolutionary mechanisms that we do not consider in our model could in theory explain those deviations, including mechanisms of gene loss that allow bacteria to eliminate detrimental genes, or selfish genetic elements (van Dijk et al., 2020). Finally, the RefSeq database is expected to contain misclassifications of contigs. This, as well as errors in genome assembly could bias the estimation of the effective HGT rate A. In addition, the representation of the various strains and taxa in the database is highly variable; this bias might affect the estimates, since our model assumes that there is a single parameter that represents the effective HGT rate between two taxa, whereas in reality the HGT rate can be different for different subtaxa/strains. In that case, the sampling bias of the database would bias the prefactor A towards the effective HGT rate of subtaxa/strains which are more represented in the database.

Although it is widely accepted that bacteria often exchange their genes with closely related species via HGT, our large-scale analysis of HGT sheds new light on gene exchange in bacteria and reveals the true scale of long-distance gene transfer events. Evidently, long-distance exchange of genetic material is a recurrent and widespread process, with specific statistical properties, suggesting that HGT plays a decisive role in maintaining the available genetic material throughout evolution.

## Materials and methods

### Identification of exact matches

Reference bacterial sequences (O'Leary et al., 2016) were downloaded from the NCBI FTP server on 3 April 2017 together with taxonomy tree files. We identified maximal exact matches using the MUMmer 3.0 (Delcher et al., 2002) software with the maxmatch option, which finds all matches regardless of their uniqueness. Specifically, to find all matches longer than 300 bp between sequences in files 1.fa and 2.fa and save it in the file Res.mumm, we used the following command:mummer -maxmatch -n -b -l 300 1.fa 2.fa > Res.mumm.

Further details can be found in the following GitHub repository: https://github.com/mishashe/HGT (Sheinman et al., 2021a, copy archived at swh:1:rev:b32b6ebd11b49349893ec69fc4788cf7ede26003, Sheinman et al., 2021b).

### Empirical calculation of the MLD for pairs of genera and sets of genera

To construct MLDs, we use all contigs longer than 10^5^ bp. The MLD of a pair of genera i and j is defined as

(3) $$m_{i\,j}\,\left(r\right)=\frac{M_{i\,j}\,\left(r\right)}{\ell_{i}\,\ell_{j}},$$

where $M_{i\,j}\,\left(r\right)$ is the number of matches of length r between all contigs of genus i and all contigs of genus j. $\ell_{x}$ is the total length of the available contigs of genus x. The expected number of matches found in the analysis of a pair of genera scales with the amount of sequence data available for these genera. Normalising by $\ell_{i}\,\ell_{j}$ therefore ensures that $m_{i\,j}\,\left(r\right)$ does not scale with the database size, so that the $m_{i\,j}\,\left(r\right)$ for different pairs of genera can be compared.

In Figure 2, Figure 5 and Figure 5—figure supplement 1, Figure 5—figure supplement 2, Figure 5—figure supplement 3, Figure 5—figure supplement 4, we show MLDs based on the matches found between pairs of sequences from two *sets* of genera. These MLDs were calculated as follows:

(4) $$m\,\left(r\right)=\frac{\sum_{i,j}m_{i\,j}\,\left(r\right)}{\sum_{i,j}1},$$

where the index i runs over the genera from the first set and the index j runs over the genera from the second set.

### Fitting the power law to the empirical data

To fit the power law (1) to the empirical data, we binned the tail (r>300) of the empirical MLD (using logarithmic binning) and then applied a linear regression with a fixed regression slope of −3 and a single fitting parameter, that is, the intercept ln⁡(A) (CalculatePrefactor.m script in the GitHub repository).

### Analytical calculation of the MLD predicted by a simple model of HGT

A simple model based on a minimal set of assumptions can account for the observed power-law distributions. We first consider a particular event of HGT in which two bacterial genera gain a long exact match of length K≫1 via HGT. After time t, the match is established in certain fractions of the populations of both genera, denoted $f_{1}$ and $f_{2}$, respectively, possibly aided by natural selection. By this time, the match is expected to be broken into shorter ones due to random mutations, which we assume occur at a constant effective rate $\mu =\left(\mu_{1}+\mu_{2}\right)/2$ at each base pair, where $\mu_{1}$ and $\mu_{2}$ are the mutation rates of genus 1 and 2.

Suppose that we now sample *n*_1_ genomes from genus 1 and *n*_2_ from genus 2 and calculate the MLD according to Equation (3). Then in the regime 1≪r<K the contribution of the matches derived from this particular HGT event is given by Ziff and McGrady, 1985; Massip and Arndt, 2013:

(5) $$m_{12}\,\left(r|t\right)=\frac{f_{1}\,n_{1}\,f_{2}\,n_{2}\,K\,{\left(2\,\mu \,t\right)}^{2}\,e^{-2\,\mu \,t\,r}}{\ell_{1}\,\ell_{2}}=\frac{f_{1}\,f_{2}\,K}{L_{1}\,L_{2}}\,{\left(2\,\mu \,t\right)}^{2}\,e^{-2\,\mu \,t\,r}.$$

Here, *L*_1_ and *L*_2_ are the average lengths of the genomes sampled from the two genera. Equation (5) shows that each individual HGT event contributes an exponential distribution to the MLD.

The full MLD is composed of contributions of many HGT events that happened at different times in the past. Assuming a constant HGT rate ρ, the HGT events are uniformly distributed over time, which results in the following full MLD (Massip et al., 2015):

(6) m12(r)=∫0∞ρm12(r|t)(dt=f1f2KL1L2ρμ1r3,

which yields the observed power law with exponent −3.

The prefactor

(7) $$A=K\,\frac{f_{1}\,f_{2}}{L_{1}\,L_{2}}\,\frac{\rho }{\mu }$$

in Equation (1) can be interpreted as an effective transfer rate per genome length. It depends on several parameters: the transfer rate from one species to another per genome length $\rho /\left(L_{1}\,L_{2}\right)$, the length of the transferred sequences K, the degree to which the sequence is establishment in the population of the two genera $f_{1}$ and $f_{2}$, and the effective mutation rate μ.

### Robustness of the power-law behaviour

For simplicity, the above argument makes several strong assumptions, including that μ, K, $f_{1}$, and $f_{2}$ are the same for all HGT events and that these events are distributed uniformly over time. However, if these assumptions are relaxed the power law proves to be remarkably robust.

First, we could assume that all of the above parameters differ between HGT events, according to some joint probability distribution $P\,\left(K,\mu ,f_{1},f_{2}\right)$. As long as this distribution itself does not depend on the time t of the event, Equation (6) then becomes

(8) $$m_{12}(r)=\iint \;\;\;\;\iint_{0}^{\infty }P(K,\mu ,f_{1},f_{2})\int_{0}^{\infty }\rho m_{12}(r|t)dt\;dK\;d\mu d\;f_{1}d\;f_{2}=\frac{\rho }{L_{1}L_{2}}\left\langle \frac{Kf_{1}f_{2}}{\mu }\right\rangle \frac{1}{r^{3}},$$

where the angular brackets denote the expectation value. The power law remains, except that the prefactor now represents an average over all possible parameter values. Second, we can relax the assumption that the divergence time t is uniformly distributed (i.e., that HGT events were equally likely at any time in the past). In general, Equation (6) should then be replaced by

(9) $$m_{12}\,\left(r\right)=\int_{0}^{\infty }P_{\text{d}}\,\left(t\right)\,\rho \,m_{12}\,\left(r|t\right)\,dt,$$

in which $P_{\text{d}}\,\left(t\right)$ is the divergence-time distribution. Previously, this distribution was assumed to equal 1, but other possibilities can be explored. For example, if instead we assume that xenologous sequences are slowly *removed* from genomes due to deletions, the divergence times may be exponentially suppressed,

(10) $$P_{\text{d}}\,\left(t\right)=e^{-\lambda \,t},$$

 in which case Equation (9) becomes:

(11) $$m_{12}\,\left(r\right)=\int_{0}^{\infty }P_{\text{d}}\,\left(t\right)\,\rho \,m_{12}\,\left(r|t\right)\,dt=\frac{f_{1}\,f_{2}\,K}{L_{1}\,L_{2}}\,\frac{\rho }{\mu }\,{\left(r+\frac{\lambda }{2\,\mu }\right)}^{-3}.$$

This MLD again has the familiar power-law tail in the regime r≫λ/(2⁢μ). Generally, if the divergence-time distribution can be written as a Taylor series

(12) $$P_{\text{d}}\,\left(t\right)=\sum_{i=0}^{\infty }\frac{a_{i}\,t^{i}}{i!},$$

Equation (9) evaluates to

(13) $$m_{12}(r)=\frac{f_{1}f_{2}K}{L_{1}L_{2}}\frac{\rho }{2\mu }\sum_{i=0}^{\infty }(i+1)(i+2)a_{i}r^{-3-i}.$$

The tail of this distribution is dominated by the first non-zero term in the series, because it has the largest exponent. Again this results in a power law with exponent −3 provided $a_{0}=P_{\text{d}}\,\left(0\right)$ does not vanish. That is, an exponent of −3 is expected provided HGT events have taken place at a non-zero rate up to the present time (Massip et al., 2015, Massip et al., 2016).

### Age-range estimation of the exact matches

According to the above model, the probability that a match of length r originates from an event that took place a time t ago is given by

(14) $$p\,\left(t|r\right)=\rho \,m_{12}\,\left(r|t\right)/m_{12}\,\left(r\right)=r^{3}\,\mu \,{\left(2\,\mu \,t\right)}^{2}\,e^{-2\,\mu \,t\,r}.$$

The most likely time $t_{ML}$ is found by setting the time derivative of Equation (14) to zero, which results in 

(15) $$t_{ML}=(\mu r{)}^{-1}.$$

Above, we considered exact matches with a length r>300 bp. Only in sequences involved in rather recent HGT events such long matches are likely to occur, and hence the method can only detect recent events. Equation (15) can provide a rough estimate for the detection horizon of the method. To do so, we substitute r=300 bp into Equation (15). Assuming a mutation rate μ of 10^−9^ per bp and per generation, this results in a detection horizon of $t_{ML}\approx 10^{6}$ generations. Assuming a mean generation time in the wild of about 10 hr (Gibson et al., 2018), this corresponds to approximately 1000 years. That is to say, we estimate that the HGT events we detect date back to the past 1000 years. We stress, however, that both the mutation rate and the generation time can strongly vary from one species to the next; hence this estimate is highly uncertain.

By Equation (15), the event that created the match of 19,117 bp in Figure 1C–D is dated back about 60 years ago, again with a large uncertainty. Vancomycin was discovered in 1952, but widespread usage started only in the 1980s, and resistant strains were first reported in 1986 (Levine, 2006).

### Restricted dataset

To quantitatively study HGT rate variations, we constructed a smaller, curated dataset to reduce the risk of potential artefacts. The curated dataset encompasses only the exact sequence matches that stem from the comparison of contigs larger than 10^6^ bp, since short contigs are more likely to present assembly or species assignment errors, or to originate from plasmid DNA. The resulting dataset comprises 138,273 matches longer than 300 bp.

Hence, using the RefSeq database, we analysed all exact sequence matches longer than 300 bp between bacteria from different bacterial families, filtering out all contigs smaller than 10^6^ bp. For some organisms we suspect an erroneous taxonomic annotation, due to their high similarity to another species, much higher than what is expected based on their reported taxonomic distances. For species for which we found very high similarity (i.e., a large number of long exact matches) to several distant species, we further compared this species to species belonging to its annotated taxa to compute the intra-taxon similarity. When the intra-taxon similarity was smaller than the similarity to distant species, we concluded that the annotation was likely erroneous. We thus manually cleaned the results, removing the comparisons between the species with suspected erroneous annotation and the taxa with which it had large similarities. Using this criterion, we removed from our database the comparison between the following accession numbers and all species of the mentioned taxa:

- Accession number NZ_FFHQ01000001.1 and all *Enterococcus*
- Accession number NZ_JOFP01000002.1 and accession number NZ_FOTX01000001.1
- Accession number NZ_LILA01000001.1’ and all *Bacillus*
- Accession number NZ_KQ961019.1’ and all *Klebsiella*
- Accession number NZ_LMVB01000001.1’ and all *Bacillus*
- Pairwise comparisons between accession numbers NZ_BDAP01000001.1, NZ_JNYV01000002.1, and NZ_JOAF01000003.1

This resulted in 138,273 unique matches.

### Environment, Gram, and GC content annotation

Ecological annotation of bacterial genera is not well defined, and different members of the same genus can occupy different ecological niches. Nevertheless, using the text mining engine of Google, we annotated some of the genera as predominately marine, gut, and soil (see paragraph 11 in the GitHub repository). Using the same approach we identified Gram-positive, Gram-negative, GC-rich, and GC-poor genera. The results are summarised in Supplementary file 7.

Additional information about bacterial genomes (such as Gram classification or lifestyle) were collected from PATRIC database metadata (Wattam et al., 2017).

### Gene enrichment analyses

To assess the enrichment of genes in the set of transferred sequences, we generated a set of control sequences as follows. For each match i present in *w*_*i*_ contigs, we randomly sampled without replacement a random sequence with the same length from each of those *w*_*i*_ contigs. This way, the control set takes into account the enrichment of certain species in the set of transferred sequences.

For the results of Figure 6 and Supplementary file 5, we analysed 12 specialised databases: acquired antibiotic resistant genes (ResFinder database; Zankari et al., 2012), antibacterial biocide and metal resistance genes database (BacMet database; Pal et al., 2014), integrative and conjugative elements (ICEberg database; Bi et al., 2012), virulence factors (VFDB database; Chen et al., 2016), essential genes (DEG database; Luo et al., 2014), toxin-antitoxin systems (TADB database; Shao et al., 2011), peptidases (MEROPS database; Rawlings et al., 2012), bacterial exotoxins for human (DBETH database; Chakraborty et al., 2012), transmembrane proteins (PDBTM database; Kozma et al., 2013), restriction enzymes (REBASE database; Roberts et al., 2015), bacterial small regulatory RNA genes (BSRD database; Li et al., 2013), the transporter classification database (TCDB; Saier et al., 2016), and enzyme classification database (Brenda; Placzek et al., 2017).

For each set of genes from a database, using the blast toolkit (Altschul et al., 1990), we calculate the total number of unique match-gene hit pairs for the matches (see paragraph 10 in GitHub repository for the exact blast command). We weighted each hit to the database by *w*_*i*_ to obtain a total number of hits H:

(16) $$H=\sum_{i}w_{i}\,n_{i}.$$

Assuming random sampling of organisms, the standard error of H is given by

(17) $$\delta \,H≃\sqrt{\sum_{i}w_{i}\,n_{i}^{2}}.$$

### SEED subsystems and GO terms ontological classification

To connect identifiers of the SEED subsystems (Overbeek et al., 2005) to accession identifiers of NCBI nr database, two databases were downloaded: nr from NCBI (NCBI Resource Coordinators, 2016) FTP and m5nr from MG-RAST (Meyer et al., 2008) FTP servers (on 17 January 2017). The homology search of proteins of the nr database against m5nr was computed using diamond v0.9.14 (Buchfink et al., 2015). Proteins from the databases were considered to have similar function if they shared 90% of amino acid similarity over the full length. Additional files for SEED subsystems (ontology_map.gz, md5_ontology_map.gz, m5nr_v1.ontology.all) were downloaded from MG-RAST FTP on the same date.

To annotate exact matches, open reading frames and protein sequences were predicted with Prodigal v2.6.3 (Hyatt et al., 2010) and queried against nr using diamond. After that subsystems classification was assigned to predicted proteins when possible.

To assign GO terms to proteins of the HGT database, we queried them against the PFAM and TIGRFAM databases using the InterProScan v5.36–75.0 (Zdobnov and Apweiler, 2001).

The scripts used to compute this analysis can be found in paragraph 6 of the GitHub repository.

The algorithms of these two systems of ontological classifications are very different. SEED subsystems is based on protein homology search with diamond, where closely related proteins will be classified within the system. Assignment of GO terms is based on HMM profiles search, where more distant relatives of proteins can be recognised.

To test for enrichment we conducted the Fisher exact test and a 95% confidence interval was obtained for the enrichment. We considered as truly enriched (resp. underrepresented) classes only the functions that were significantly enriched (resp. depleted) in both GO and SEED functional analyses. For further details, see the code in the GitHub repository (Enrichment.R).

## Data Availability

Results of the analysis are provided as supplementary files.

## Additional files

Supplementary file 1. The estimate of the effective horizontal gene transfer (HGT) rate (A) for all pairs of families.

Supplementary file 2. The estimate of the effective horizontal gene transfer (HGT) rate (A) for all pairs of genera.

Supplementary file 3. Cumulative genome length per family.

Supplementary file 4. Number of genomes with at least one long-distance horizontal gene transfer (HGT) per taxa and the cumulative length of all exact matches found for each taxa.

Supplementary file 5. Enrichment of the horizontal gene transfer (HGT) sequences for different functional classes for the 12 queried databases (see Gene enrichment analyses).

Supplementary file 6. Results of the functional enrichment analyses (SEED subsystems and gene ontology [GO] term analysis).

Supplementary file 7. GC, Gram, and environmental annotations for each family.

Supplementary file 8. DNA sequence of the two longest matches found between an archaea and bacteria.

Supplementary file 9. Matlab code to calculate the horizontal gene transfer (HGT) rate (*A*) from the comparison of a pair of genera computed with the MUMmer software.

Transparent reporting form

## Appendix 1

Phylogenetic analysis among HGT event in *E. coli* If an HGT event happened between a species of taxon A and the common ancestor of species B and B’ before the evolutionary splits of B and B’, we expect to observe two HGT events (e.g., between A and B and between A and B’). Indeed, we find that closely related strains often have exactly the same matches to distant families. As shown in Figure 1—figure supplement 2, pairs of *E. coli* strains with high average nucleotide identity (ANI) tend to share such matches. However, this effect is not very strong, probably because ANI does not accurately reflect the evolutionary distance between pairs of strains. To investigate this further, we retrieved the sequence of each match between *E. coli* strain and a species outside the *Escherichia* family. We then aligned all those matches (with blast) to all other *E. coli* strains and kept all alignments with an *E*-value $<10^{-5}$ to assess, for each HGT event, its presence in each *E. coli* strain. The resulting matrix is shown in Figure 1—figure supplement 3. One can see that *E. coli* strains cluster to compact groups, possibly reflecting their phylogeny (see Figure 1—figure supplement 3 (a)). The abundance of matches among *E. coli* strains exhibits a bi-modal distribution (see Figure 1—figure supplement 3 (b)), possibly indicating the direction of HGT: matches that are abundant in *E. coli* have likely been transferred from *E. coli* to the distant family, whereas matches that are rare in *E. coli* were likely transferred from the distant family to *E. coli* . As shown in Figure 1—figure supplement 4, sharing a match to a different family (within a blast hit) is not strongly related to ANI distances; the association can be detected only statistically, as mentioned in Figure 1—figure supplement 2. Comparing bacterial and archaeal genomes It is known that bacteria and archaea have exchanged genetic material during their evolution (Aravind et al., 1998; Nelson et al., 1999; Garcia-Vallvé et al., 2000). However, comparing all bacterial and archaeal RefSeq contigs longer than 10^6^ bp, we find only several exact matches longer than 300 bp. The longest one is of length 727 (see sequence 1 in Supplementary file 8). This exact sequence exists in archaeon *Methanobacterium* sp. *MB1* and two bacteria: *Mahella australiensis 50–1 BON* and *Petrotoga mobilis SJ95*. The amino acid sequence of this match hits the (2Fe-2S)-binding protein, known to exist in both the bacterial and archaeal kingdoms. *Mahella australiensis* gen. nov., sp. nov. (phylum: *Firmicutes*) is a moderately thermophilic anaerobic bacterium isolated from an Australian oil well (Bonilla Salinas et al., 2004). *Petrotoga mobilis* (phylum: *Thermotogae*) bacteria appear to be common members of the oil well microbial community. Petroleum reservoirs are a unique subsurface environment characterised by high temperatures, moderate to high salt concentrations, and abundant organic matter (Hu et al., 2016). *Methanobacterium* sp. *Mb1*, a hydrogenotrophic methanogenic archaeon, was isolated from a rural biogas plant producing methane-rich biogas from maize silage and cattle manure in Germany (Maus et al., 2013). We found a blast hit with more than 99% identity to this match in nine other species: *Pseudothermotoga elfii DSM 9442*, *Clostridium clariflavum DSM 19732*, *Fervidobacterium pennivorans strain DYC*, *Thermoanaerobacter* sp. *X513*, *Thermoanaerobacter* sp. *X514*, *Fervidobacterium pennivorans DSM 9078*, *Thermoanaerobacterium thermosaccharolyticum DSM 571*, and *Fervidobacterium islandicum strain AW-1*. Just next to this match the same species share another match of length 496 (see sequence 2 in Supplementary file 8.fa). This match codes for signal peptidase II, also known to exist in both bacterial and archaeal kingdoms and, probably, plays some role in an antibiotic resistance (Xiao and Wall, 2014). Enrichment of gene functions in HGT

**Appendix 1—table 1.** Enrichment of different gene categories relative to the control set (see Gene enrichment analyses in Materials and methods).

Database	Enrichment
Antimicrobial resistance, Zankari et al., 2012	36.6±0.2
Biocide and metal resistance, Pal et al., 2014	3.6±0.03
Restriction enzymes, Roberts et al., 2015	0.34±0.01
Transmembrane proteins, Kozma et al., 2013	
α	0.6±0.02
β	0.98±0.07
Peptidases, Rawlings et al., 2012	0.084±0.004
Exotoxins, Chakraborty et al., 2012	0.006±0.003
Integrative, conjugative, Bi et al., 2012	23.9±0.1
Virulence factors, Chen et al., 2016	1.2±0.026
Essential genes, Luo et al., 2014	0.23±0.002
Small regulatory RNAs, Li et al., 2013	0.01±0.005
Toxin-antitoxin, Shao et al., 2011	9.5±0.6
Transport proteins, Saier et al., 2016	
Channels/pores	0.32±0.01
Electrochemical transporters	0.56±0.01
Primary active transporters	0.96±0.01
Group translocators	0.018±0.002
Transmembrane electron carriers	0.026±0.004
Accessory factors	0.08±0.01
Incompletely characterised	0.24±0.01
Enzymes, Placzek et al., 2017	
Isomerases	0.61±0.001
Hydrolases	0.39±0.0004
Ligases	0.35±0.001
Oxidoreductases	0.33±0.0004
Transferases	0.33±0.0004
Lyases	0.16±0.0003
