# Supplementary material for: Identical sequences found in distant genomes reveal frequent horizontal transfer across the bacterial domain
Source: eLife. 2021 Jun 14;10:e62719. doi: 10.7554/eLife.62719 (PMC8270642; doi:10.7554/eLife.62719)
Supplement: Peer review history

# Decision letter

**DOI:** 10.7554/eLife.62719.sa1

**Affiliations:** University of BaselSwitzerland; University of BaselSwitzerland

In the interests of transparency, eLife publishes the most substantive revision requests and the accompanying author responses.

**Acceptance summary:**

Sheinman and colleagues investigate long stretches of identical sequences found in genome assemblies of bacteria across genera. They show that such shared identical sequence fragments exist even between evolutionarily very distant species and that the length distribution of these stretches follows a universal form given by an inverse third power. This dependence can be explained by a model assuming that identical stretches originating from horizontally transferred segments are broken up into smaller fragments by point mutations. This original approach to an important topic in bacterial evolution has to potential to shed new light on prokaryotic diversity.

**Decision letter after peer review:**

Thank you for submitting your article "Identical sequences found in distant genomes reveal frequent horizontal transfer across the bacterial domain" for consideration by *eLife*. Your article has been reviewed by 3 peer reviewers, including Richard A Neher as the Reviewing Editor and Reviewer #1, and the evaluation has been overseen by Gisela Storz as the Senior Editor.

The reviewers have discussed the reviews with one another and the Reviewing Editor has drafted this decision to help you prepare a revised submission.

As the editors have judged that your manuscript is of interest, but as described below that additional analyses are required before it is published, we would like to draw your attention to changes in our revision policy that we have made in response to COVID-19 (https://elifesciences.org/articles/57162). First, because many researchers have temporarily lost access to the labs, we will give authors as much time as they need to submit revised manuscripts. We are also offering, if you choose, to post the manuscript to bioRxiv (if it is not already there) along with this decision letter and a formal designation that the manuscript is "in revision at *eLife*". Please let us know if you would like to pursue this option.

Summary:

Sheinman and colleagues investigate long stretches of identical sequences found in genome assemblies of bacteria across genera. They show that such shared identical sequence fragments exist even between evolutionarily very distant species and that the length distribution of these stretches follows a universal form given by an inverse third power. This dependence can be explained by a model assuming that identical stretches originating from horizontally transferred segments are broken up into smaller fragments by point mutations. The inferred rate of exchange shows systematic patterns and is higher between taxonomically closer species.

Essential revisions:

All reviewers agreed that this manuscript presents an interesting and original approach to an important topic in bacterial evolution, but raised a number of concerns regarding the robustness of the results that need to be addressed and suggested how the presentation of the results could be improved.

1) Robustness to phylogenetic correlations and biased sampling. A heavily sampled species might contain many vertically inherited identical stretches that will be compared to each other and to a potential horizontal transfer in a different species. Such correlations and oversampling can therefore inflate the estimated transfers. It is hence critical that the authors investigate and discuss how their conclusions depend on phylogenetic relationships, diversity, and sampling density in their data set.

2) Plasmids and assembly artefacts: The manuscript contains a brief section on a restricted data set encompassing only contigs longer than 1 Mb to effectively exclude plasmids and misassigned short contigs. In this data set, the authors also find long-distance HGT. But it remains unclear whether the statistical patterns described for the full data set are consistent with this smaller data set. We would like to see a quantitative assessment of the contribution of plasmids and assembly artefacts. Furthermore, you seem to sometimes find identical sequences fragments longer then 300 kb shared between distant species. These should be spot-checked.

3) The enrichment analysis for functional categories should be done more systematically. It is by now routine to assign genes in genomes to Gene Ontology categories (e.g. using Interpro or Pfam) in a systematic manner, which would give statistics for all gene ontology categories.

4) The 1/r^3^ dependence of match length distribution is central part of the analysis and needs to be better explained. Currently, it is left to reader to go through the math in the methods section, look up the reference, and develop an intuition for the behavior. A discussion (and possibly illustration) of the process through which the 1/r^3^ distribution emerges would make the manuscript much more self-contained and help the reader to assess the robustness of the conclusions.

Reviewer #2:

I just have one main comment. This calculation of the probability of obtaining the exact DNA match in a pair of genomes (lines 46-48) obviously does not account for selection. If we are dealing with a highly conserved genetic region (eg, a ribosomal RNA protein), the chances of observing an identical DNA fragment are going to be much higher different. According to my rough calculations, they should be of the order of 1e-10, hence it still shouldn't really affect the results, but it took me a while to come to this conclusion. I would ask the authors to make it more explicit in the article why the trick of looking at exact matches overcomes this problem.

[Editors' note: further revisions were suggested prior to acceptance, as described below.]

Thank you for resubmitting your work entitled "Identical sequences found in distant genomes reveal frequent horizontal transfer across the bacterial domain" for further consideration by *eLife*. Your revised article has been evaluated by Gisela Storz (Senior Editor) and a Reviewing Editor.

The manuscript has been improved and you have addressed most concerns the were raised in the review but there are some remaining issues that need to be addressed, as outlined below:

1) Please explain the abbreviations G/F, F/O etc.

2) The methods need more documentation. You provide a single matlab script without comments and hard coded paths that only covers a fraction of the analysis. This is not an acceptable standard of reproducibility for a computational biology paper.

– What are the exact options Mummer was run with? On what set of genomes?

– Scripts need commenting and explanation what input they take and what output they produce.

– Enrichment analysis use a number of tools, but no details are given how exactly they are run and how the output is processed into the figures and tables in the manuscript.

# Reply: Author response

**DOI:** 10.7554/eLife.62719.sa2

Essential revisions:

All reviewers agreed that this manuscript presents an interesting and original approach to an important topic in bacterial evolution, but raised a number of concerns regarding the robustness of the results that need to be addressed and suggested how the presentation of the results could be improved.

We thank the editor and reviewers for their constructive criticism, which has helped us to significantly improve the manuscript. In response to the reviewers’ comments, we have notably added 7 supplementary figures (figure 1-supplementary figure 1 to 4, figure 2 - supplement figure 1 and 2 and figure 3 -supplement figure 1). We have also added a detailed explanation of the evolutionary model (as Box 1) and 2 supplementary sections giving details about the additional analysis we have performed. Finally, we have modified the text in accordance with the reviewers’ comments.

Below, we also provide a point by point answer to the reviewer's comments.

1) Robustness to phylogenetic correlations and biased sampling. A heavily sampled species might contain many vertically inherited identical stretches that will be compared to each other and to a potential horizontal transfer in a different species. Such correlations and oversampling can therefore inflate the estimated transfers. It is hence critical that the authors investigate and discuss how their conclusions depend on phylogenetic relationships, diversity, and sampling density in their data set.

Indeed, even the curated RefSeq database is biased and the representation of strains/taxa differs from one strain/taxa to the other.

First, however, we would like to point out that we do not, in any of our analyses, compare genomes of species that belong to the same genus, precisely to avoid the issue that “many vertically inherited identical stretches (…) will be compared to each other”, as pointed out by the reviewers. We have highlighted this point l. 89-90 in the revised version of the manuscript.

Second, we stress that we defined the normalization of the match-length distributions precisely to ensure that the prefactor does *not* scale with the total amount of sequence information for each of the taxa in the database. The MLD for a pair of genera i and j is defined as $m_{\text{ij}}=\;\frac{M_{\text{ij}}(r)}{l_{i}l_{j}},$ where $M_{\text{ij}}$ is the number of matches of length r between all contigs of genus i and all contigs of genus j. Crucially, the MLD is normalized by the product of $l_{i}$ and $l_{j}$, representing the total length of the available sequences of genera i and j. If the number of genomes in one of the genera is increased, the expectation of both the numerator and the denominator grows (linearly), and hence the MLD is not expected to change. Similarly, if one would artificially increase the number of genomes in the database for a taxon by randomly adding already existing ones, the expectation of the prefactor A would not change because we normalize the MLD by the total length of the genomes. Thus, the problem that “many vertically inherited identical stretches (..) will be compared (…) to a potential horizontal transfer in a different species” is avoided.

One can appreciate that the prefactor does not increase with the (average) total genome length of family pairs in Author response image 1.

**Author response image 1.** The prefactor A versus the (average) total genome length of pairs of families.

That being said, we do agree that sampling bias in the database might still affect our estimates in a different way. Our model summarises the effective HGT rate between two taxa with a single number, whereas in reality the rate can be different for different subtaxa/strains. This, combined with a biased representation of different subtaxa/strains in the database, can bias the prefactor A (effective HGT rate) towards the effective HGT rate of subtaxa/strains that are more represented in the database. To make this point clearer, consider the extreme situation of a taxon for which a single strain is sequenced many times and overwhelms the database for this taxon. In this case the estimated prefactor for this taxon will be biased towards the value relevant only for this single strain.

There is, however, no good way to correct for this bias, since it would require manual curation of the extremely large database and would drastically reduce the power of our analysis. (Besides, to do so, one would have to know what the “correct” weight of each genome would be within the taxum.)

On the other hand, if the rate of HGT between the two taxa is similar for their subtaxa/strains, the value of A does not depend on the sampling properties. Moreover, the power-law distribution (and its -3 exponent) is robust to sampling bias and we see it, as we expected, for many different taxa comparisons.

We have clarified this point in the revised version of the manuscript, l. 421-429 to highlight this limitation of our method.

2) Plasmids and assembly artefacts: The manuscript contains a brief section on a restricted data set encompassing only contigs longer than 1 Mb to effectively exclude plasmids and misassigned short contigs. In this data set, the authors also find long-distance HGT. But it remains unclear whether the statistical patterns described for the full data set are consistent with this smaller data set. We would like to see a quantitative assessment of the contribution of plasmids and assembly artefacts. Furthermore, you seem to sometimes find identical sequences fragments longer then 300 kb shared between distant species. These should be spot-checked.

We agree with the reviewer that assembly artefacts could impact our estimates of the transfer rates.

To assess the impact of such artefacts on our model, we constructed the curated set in the following way. First of all, only matches between contigs larger than 10^6^ bp were included in this restricted dataset. Because assembly quality increases with contig size and because almost all plasmids are shorter than 10^6^ bp, the curated set is expected to be devoid of most assembly artefacts and plasmids. Second, to be able to manually examine the matches, we considered only matches from species belonging to different families (the full database including comparisons between species of the same families but different genera is too large to allow manual curation). Third, because the restricted set is much smaller than the full database, we were able to manually examine all very long matches. Doing so, we discovered that the phylogenetic annotations of some contigs were incorrect and removed all the erroneous comparisons from the database. We further clarified this point in the manuscript l. 168-177.

Although the restricted set has less statistical power than the full set, it can be used to check the validity of our results. As Figure 2-supplement 1 in the revised version of the manuscript, we reproduce the same MLDs as presented in Figure 2 but now using the curated set rather than the full set. (No comparison between *Escherichia* and *Klebsiella* is shown because these two species belong to the same family). While the number of matches observed in the curated set is much smaller, the distributions we obtain present a good fit to the -3 power law. In addition, the estimates of the prefactors A are close to (of the same order of magnitude as) the prefactors calculated with the full set (see Figure 2). These analyses show that the estimates of A computed based on the full set are robust to assembly artefacts and erroneous annotations and are not driven by plasmids exchanges.

We added a figure (Figure 2-supplement 1) to the manuscript and we discuss this analysis in the result section l. 168-177 and 207-209 of the revised manuscript.

3) The enrichment analysis for functional categories should be done more systematically. It is by now routine to assign genes in genomes to Gene Ontology categories (e.g. using Interpro or Pfam) in a systematic manner, which would give statistics for all gene ontology categories.

We thank the reviewers for this suggestion. We would first like to point out that our initial analysis with the SEED Subsystems is also systematic, since each individual protein found on one of the exact matches was annotated via the SEED subsystems, a popular ontology that classifies all proteins on three levels with increasing functional resolution and specificity.

We opted for the SEED subsystems because it provides three levels of functional groupings, which facilitate the biological interpretation. We have clarified this point and the difference between the SEED and GO strategies in the method section (Method section, “SEED subsystem and GO terms ontological classification” l. 566-588).

To ascertain our results, however, we have now added a GO-term analysis to the revised version of the manuscript. Overall, we found a very good agreement between the two methods for most categories, showing that our results are well supported. We note, however, that the GO analysis did not confirm the enrichment for genes of the flagellum category that we previously reported based on the SEED analysis; we therefore decided to remove this result from the manuscript. Instead, the GO term analysis highlighted the enrichment for proteins of Secretion systems and proteins involved in Sporulation, which are both supported by the Seed Subsystem analysis. We describe those new results l. 331-366 of the revised version of the manuscript.

4) The 1/r^3^ dependence of match length distribution is central part of the analysis and needs to be better explained. Currently, it is left to reader to go through the math in the methods section, look up the reference, and develop an intuition for the behavior. A discussion (and possibly illustration) of the process through which the 1/r^3^ distribution emerges would make the manuscript much more self-contained and help the reader to assess the robustness of the conclusions.

Thank you for this suggestion. To facilitate the understanding of the emergence of the -3 power law, we have now added a schematic illustration together with a short and simplified explanation of the model to the revised manuscript, as Box 1.

Reviewer #2:

I just have one main comment. This calculation of the probability of obtaining the exact DNA match in a pair of genomes (lines 46-48) obviously does not account for selection. If we are dealing with a highly conserved genetic region (eg, a ribosomal RNA protein), the chances of observing an identical DNA fragment are going to be much higher different. According to my rough calculations, they should be of the order of 1e-10, hence it still shouldn't really affect the results, but it took me a while to come to this conclusion. I would ask the authors to make it more explicit in the article why the trick of looking at exact matches overcomes this problem.

We thank the reviewer for this comment. We have replaced the calculation, considering the most conserved regions of genomes (rRNA, at most 90% identity for species from different genera, see https://jb.asm.org/content/196/12/2210, Figure 3). This gives us a probability to find a match of 300bp of the order of 10^-14^. We have also made the calculation more explicit in the revised version of the manuscript (l. 97).

[Editors' note: further revisions were suggested prior to acceptance, as described below.]

The manuscript has been improved and you have addressed most concerns the were raised in the review but there are some remaining issues that need to be addressed, as outlined below:

We want to thank the editor and reviewers again for their constructive criticism to improve the reproducibility of our work. As suggested, we have clarified the caption of figure 5, we made a github repository (https://github.com/mishashe/HGT) containing all the scripts used for our analysis and have clarified the method section.

Below, we provide more detailed answers to the reviewer's comments.

1) Please explain the abbreviations G/F, F/O etc.

On Figure 5, we present the match length distributions computed from subsets of comparisons, in order to compare species at given taxonomic distances from each other. G/F stands for “family but not genera” ie: we compare together all species belonging to the same family, excluding all comparisons between species belonging to different genera.

F/O is the same, but for a higher phylogenetic level: we compare all species that belong to the same order, excluding comparisons between species that belong to the same family. And so on:

O/C: species of the same class excluding species of the same Order

C/P : species of the same phyla, but different class.

P/D: species of the same domain, but different phyla.

We have modified the caption of figure 5 to clarify this point

2) The methods need more documentation. You provide a single matlab script without comments and hard coded paths that only covers a fraction of the analysis. This is not an acceptable standard of reproducibility for a computational biology paper.

– What are the exact options Mummer was run with? On what set of genomes?

– Scripts need commenting and explanation what input they take and what output they produce.

– Enrichment analysis use a number of tools, but no details are given how exactly they are run and how the output is processed into the figures and tables in the manuscript.

We thank the reviewers for their suggestion and apologize for the low level of details provided in the previous version. We have created a github repository containing scripts to reproduce our analyses. We have also modified the Method section to improve the reproducibility and to point to precise scripts in the github repository for each of the analyses. The github repository is accessible here: https://github.com/mishashe/HGT
